# Development and Feasibility of an App to Decrease Risk Factors for Type 2 Diabetes in Hispanic Women With Recent Gestational Diabetes (Hola Bebé, Adiós Diabetes): Pilot Pre-Post Study

**DOI:** 10.2196/19677

**Published:** 2020-12-31

**Authors:** Ellen W Seely, Patricia Flynn Weitzman, Dharma Cortes, Sara Romero Vicente, Sue E Levkoff

**Affiliations:** 1 Endocrinology, Diabetes and Hypertension Division Brigham and Women's Hospital Harvard Medical School Boston, MA United States; 2 Environment and Health Group Cambridge, MA United States; 3 University of South Carolina Columbia, SC United States

**Keywords:** gestational diabetes mellitus, diabetes prevention, Hispanic, Latina, intervention, mobile apps, mHealth

## Abstract

**Background:**

Hispanic women have increased risk of gestational diabetes mellitus (GDM), which carries an increased risk for future type 2 diabetes, compared to non-Hispanic women. In addition, Hispanic women are less likely to engage in healthy eating and physical activity, which are both risk factors for type 2 diabetes. Supporting patients to engage in healthy lifestyle behaviors through mobile health (mHealth) interventions is increasingly recognized as a viable, underused tool for disease prevention, as they reduce barriers to access frequently experienced in face-to-face interventions. Despite the high percentage of smartphone ownership among Hispanics, mHealth programs to reduce risk factors for type 2 diabetes in Hispanic women with prior GDM are lacking.

**Objective:**

This study aimed to (1) develop a mobile app (¡Hola Bebé, Adiós Diabetes!) to pilot test a culturally tailored, bilingual (Spanish/English) lifestyle program to reduce risk factors for type 2 diabetes in Hispanic women with GDM in the prior 5 years; (2) examine the acceptability and usability of the app; and (3) assess the short-term effectiveness of the app in increasing self-efficacy for both healthy eating and physical activity, and in decreasing weight.

**Methods:**

Social cognitive theory provided the framework for the study. A prototype app was developed based on prior research and cultural tailoring of content. Features included educational audiovisual modules on healthy eating and physical activity; personal action plans; motivational text messages; weight tracking; user-friendly, easy-to-follow recipes; directions on building a balanced plate; and tiered badges to reward achievements. Perceptions of the app’s acceptability and usability were explored through four focus groups. Short-term effectiveness of the app was tested in an 8-week single group pilot study.

**Results:**

In total, 11 Hispanic women, receiving care at a federally qualified community health center, aged 18-45 years, and with GDM in the last 5 years, participated in four focus groups to evaluate the app’s acceptability and usability. Participants found the following sections most useful: audiovisual modules, badges for completion of activities, weight-tracking graphics, and recipes. Suggested modifications included adjustments in phrasing, graphics, and a tiering system of badges. After app modifications, we conducted usability testing with 4 Hispanic women, with the key result being the suggestion for a “how-to tutorial.” To assess short-term effectiveness, 21 Hispanic women with prior GDM participated in the pilot. There was a statistically significant improvement in both self-efficacy for physical activity (*P*=.003) and self-efficacy for healthy eating (*P*=.007). Weight decreased but not significantly. Backend process data revealed a high level of user engagement.

**Conclusions:**

These data support the app’s acceptability, usability, and short-term effectiveness, suggesting that this mHealth program has the potential to fill the gap in care experienced by Hispanic women with prior GDM following pregnancy. Future studies are needed to determine the effectiveness of an enhanced app in a randomized controlled trial.

**Trial Registration:**

ClinicalTrials.gov NCT04149054; https://clinicaltrials.gov/ct2/show/NCT04149054

## Introduction

Gestational diabetes mellitus (GDM), defined as glucose intolerance diagnosed after the first trimester of pregnancy [1], occurs in 3%-7% of pregnancies in the United States. Due, in part, to the fact that 40% of Hispanic women in the United States of child-bearing age are obese, and 51% experience excessive weight gain during pregnancy [2-5], this group has 1.5 times the risk of GDM compared to non-Hispanic White women [6]. GDM carries an overall increased risk as high as 60% for the development of type 2 diabetes mellitus (T2DM) [7], placing Hispanic women with prior GDM at high risk for future T2DM. Furthermore, obesity, a major risk factor for GDM and the strongest modifiable risk factor for T2DM, is more prevalent among Hispanic than non-Hispanic White women [8,9].

It is widely acknowledged that Hispanic women in the United States experience disparities in health care access and utilization compared to non-Hispanic women [10]. Cultural, social, and economic barriers also lead to disparities in healthy lifestyle behaviors [10]. Hispanic women face sociocultural barriers to healthy eating (eg, cost of healthy food, knowledge about nutritional values of some foods, and family food preferences) [11], as well as structural barriers (eg, food deserts) to obtaining healthy foods [12]. Hispanic women are also less likely to be physically active compared to non-Hispanic White women [13-15]. While some barriers to physical activity are comparable to those for non-Hispanic women (eg, lack of time, lack of childcare, being tired, and having limited self-discipline) [13]. Some additional barriers may be culturally influenced such as being discouraged by family members and friends, as well as environmental factors, such as not having a safe place to exercise [16].

The Diabetes Prevention Program (DPP), which was delivered as a face-to-face intervention, demonstrated that T2DM can be prevented by lifestyle changes focused on healthy eating and physical activity in women with a remote history of self-reported GDM [17]. The scalability of face-to-face DPP-based programs has been a challenge, due to the costs involved in implementing an in-person intervention and the difficulties encountered in attending face-to-face programs [18,19], particularly in postpartum women with recent GDM [20]. A potential approach to overcome barriers to face-to-face implementation is through mobile health (mHealth) technologies that can enable greater patient access.

According to the Pew Research Center, approximately 80% of the Hispanic population owns a smartphone, which is comparable to White and Black populations [21], with Hispanics more likely to use their smartphone to seek health information than their White counterparts [22]. Hispanic people in the United States spend more time using apps than the general population [23]. These data suggest that mobile apps are a viable, underused tool for T2DM prevention in minority populations including Hispanic women with recent GDM.

These findings led us to develop and pilot test a culturally tailored, bilingual (Spanish/English), mobile app–based lifestyle program, *¡Hola Bebé, Adiós Diabetes!* (hereafter referred to as Hola Bebé), to reduce risk factors for T2DM in Hispanic women who have had GDM in the prior 5 years. The years after childbirth are well recognized as representing a “window of opportunity” to improve the future health of women who have had GDM, as demonstrated in our previous work [24,25] and by other studies [26,27]. The goal of the Hola Bebé pilot was to determine the feasibility, acceptability, and short-term effectiveness of an mHealth approach to increasing self-efficacy for healthy eating and increased physical activity, and promoting weight loss, in a population of Hispanic women with recent GDM.

## Methods

### Overview

Social cognitive theory (SCT) provided the framework for the Hola Bebé intervention. Self-efficacy, the belief in one’s own capabilities to adopt and maintain behavior change [28,29], is a core component of SCT. For the intervention, we developed educational and motivational messages delivered through texts and videos to increase self-efficacy for healthy diet and physical activity. The focus on healthy eating and physical activity for the app was based on the DPP, which demonstrated that lifestyle change targeting healthy eating and increased physical activity led to a decrease in the development of T2DM in individuals at high risk for this condition including women with prior history of GDM [17]. Motivational messages were developed to target self-efficacy, which is associated with initiation and adherence to physical activity and other health-promoting activities [30,31]. Participants chose the times of day and frequency of the text messages. Cultural tailoring involved the development of the app first in Spanish, followed by translation into English with input from Hispanic women with a history of GDM who participated in every stage of app development.

**Figure 1:**
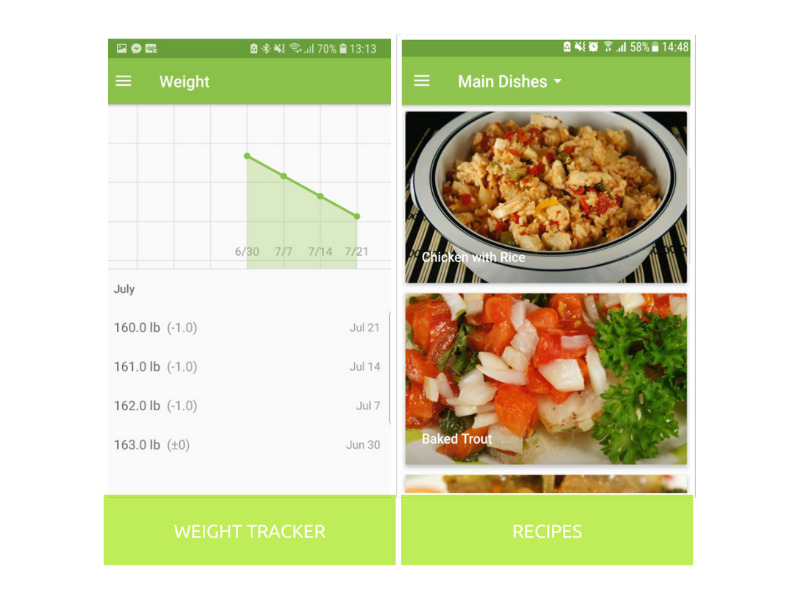
Screenshots from the ¡Hola Bebé, Adiós Diabetes! app.

The app included six educational audiovisual modules on healthy eating and physical activity; personal action plans for healthy eating and staying active; motivational and educational text messages; weight tracking; user-friendly, easy-to-follow recipes (Figure 1); directions on how to build a balanced plate; and tiered badges to reward achievements. For the action plans, participants were taught how to identify barriers to individualized goals and ways to overcome the barriers. Healthy eating advice was based on MyPlate [32]. Tiered badges could be earned by the participants with completion of a module, action plan, and/or inputting of weight. The app was developed to meet the 8th-grade literacy level. All content was in plain-language Spanish and English, with Spanish and English audio voiceover.

### Formative Phase

In our formative work, we solicited feedback on the acceptability and usability of the mHealth program through four focus groups. Inclusion criteria included Hispanic women, aged 18-45 years, with prior GDM in the past 5 years, and who received medical care at a federally qualified community health center (CHC), a Level 3 Patient-Centered Medical Home in the Greater Boston area. In total, 11 women participated in the acceptability focus groups. Participants were asked for feedback on the prototype of the app, including what feature(s) they found most useful, preferences for phrasing, wording, graphics, colors, and type of badge tier system. The sections they found most useful were (1) the audiovisual modules, especially those about how to make healthy choices when eating out; (2) the badges for completion of activities; (3) the weight tracking graphic; and (4) the recipes. They also recommended a color scheme from a menu of options and offered suggestions for certain adjustments in phrasing and graphics. Finally, they suggested that the tiering system of badges be based on a system of student achievement, such as “outstanding student” or “honor student.”

After modifications to the prototype app were made based on participants’ input, we conducted usability testing with 4 participants. Participants were given access to the app and asked to perform a number of tasks (eg, click on a tab, complete a module, and open an action plan tab), as well as to explore as they wished. The key result from the usability testing was that participants requested a “how-to tutorial” to make the app easier to use. Some, in fact, offered specific suggestions, such as “click here to add your weight.” Participants also asked for more tabs to better label and access specific sections of the app. In addition, for the action plan completion section, participants suggested instead of only providing a free-text box, there be an additional drop down menu of action plan options with prepopulated action plans to choose from (eg, “I will balance my plate at dinner,” “I will eat fruit and/or vegetable with every meal”). Feedback on the acceptability and usability was incorporated in the app prior to the pilot (Table 1).

**Table 1:** Features of the ¡Hola Bebé, Adiós Diabetes! app.

Domains	Features
Healthy eating education and tools	Healthy eating educational audiovisual modules Healthy recipes MyPlate education and demonstration Action plans for healthy eating
Physical activity education and tools	Physical activity educational audiovisual modules Exercise videos Action plans to stay active
Text messaging	Automated motivational text messages for healthy eating Automated motivational text messages for physical activity
Self-monitoring	Weight tracking Gain badges by completing educational modules and action plans
Social network	Sharing of experiences and recipes among participants through a community forum Asking questions of other participants through a community forum
Operating system	Android

### Pilot Trial

A nurse and a medical assistant from the health center identified potential participants who were Hispanic and who had had GDM in the past 5 years from a list generated from the CHC database using the same inclusion criteria as in the formative phase. In addition, participants had to have or be willing to use an Android mobile phone for the study. For women with other smartphones, Android phones were offered on loan for the duration of the study. A member of the study team contacted those women who expressed interest in participating to provide additional information about the study. For those women who agreed to participate, the research assistant scheduled the first study visit. At the first study visit, informed written consent was obtained, and the participant’s weight and height were determined by the research assistant. Weight was measured with the DR400C/Detecto Portable Home Health Care Scale, which was zeroed prior to each weight determination, with the participant wearing light clothes. Participants were asked to complete self-efficacy questionnaires for healthy eating (20 items) and physical activity (12 items) developed by Sallis et al [33], scored on a scale of 1-5 with 5 being the most self-efficacious. These questionnaires have been widely used in research in both Spanish [34] and English. A research assistant helped the participants download (via the Google Play store) and open the app on their phone, and review the “how to use the app” tutorial. Participants were asked to watch one module weekly for the 8 weeks of study duration, complete the corresponding action plan, weigh themselves, and enter their weight into the app. At the end of the 8 weeks, baseline measures were repeated, and structured exit interviews, which focused on what participants liked best and areas for improvement, were performed. Primary outcomes included self-efficacy for healthy eating and self-efficacy for physical activity, with weight as a secondary outcome.

The study was approved by the Pearl Institutional Review Board and the board of the CHC. All participants signed written informed consent.

### Statistical Analysis

Descriptive statistics were presented as mean (SD) and frequency (%). For pre-post comparisons from the pilot study, paired *t* tests were conducted with a 5% significance level.

## Results

In total, 30 eligible women were identified from the CHC database; 4 women could not be contacted. Of this, 26 women were successfully notified about the study and 21 (88%) consented to participate. Reasons given for not participating included not being interested in participating (n=3), moving out of state (n=1), and not wanting to use a study-provided Android phone (n=1) (Table 2). At baseline, 21 participants were assessed and 18 completed the 8-week study.

**Table 2:** Baseline characteristics of the study population (N=21).

Characteristic		Value
Age (years), mean (SD)		33 (6.9)
Number of pregnancies, mean (SD)		3 (1.83)
Number of pregnancies with GDM^a^, mean (SD)		1 (0.57)
Years post-GDM pregnancy, mean (SD)		2.9 (0.74)
**Smartphone operating system, n (%)**		
	Android	14 (66.6)
	iOS	7 (33.3)
Family history of diabetes in first-degree relative, n (%)		14 (66.6)
Ethnicity: Hispanic, n (%)		21 (100)
Primary language: Spanish, n (%)		21 (100)

^a^GDM: gestational diabetes mellitus.

### Short-Term Effectiveness

Self-efficacy for healthy eating increased from 4.2 (SD 0.8) to 4.4 (0.7) (*P*=.007). Self-efficacy for physical activity increased from 3.0 (SD 0.6) to 3.4 (SD 0.6) (*P*=.003). The secondary outcome, weight, fell from 163 (SD 36) to 162 (SD 37) lbs (*P*=.16).

### Engagement

Backend process data revealed a high level of user engagement. In total, 91% (19/21) of participants viewed audiovisual modules and created action plans. There was also a high level of engagement in earning badges, with 95% of participants (20/21) earning badges by completing a learning module and/or an action plan or weighing. Participants posted tips on the community forum for other participants, such as a family recipe, and asked questions that other participants answered. One woman did not participate in any of the trackable app features.

### Exit Interviews

We conducted exit interviews at the conclusion of the pilot study. The following quotes are representative of the participants’ experiences of using the app:

No cambiaría nada de la aplicación, me gusta todo (I would not change anything from the app, I like everything).

Los videos de los módulos me han ayudado a entender la clase de alimentos que son buenos para mi. He comenzado a cambiar los granos por granos integrales y ahora me siento más saludable (The module videos helped me understand what kind of foods were good for me. I started changing my grains for whole wheat grains and now I feel healthier).

Cuando voy a comer con mi familia a un restaurante, ya sé que clase de comida puedo ordenar y no sentirme culpable después (Whenever I go to eat with my family to a restaurant, I know which kind of food I can order and not feel guilty afterwards).

Participants especially liked the personalized action plans, the motivational text messages, the at-home exercise videos, and the recipes. Women commented that they found the “how to use the app” tutorial to be helpful.

Participants had suggestions for incorporation in a future version of the app. They requested more exercise videos including Zumba and with the addition of music, expansion of the recipe section to include more Latin American dishes and vegetarian options, and explanation of portion sizes for each recipe that align with MyPlate. Women also requested videos for recipe preparation. Participants asked for an “ask the expert” option to submit specific exercise and diet questions on the community forum. The 7 iPhone users asked that an app be developed for use on an iPhone.

## Discussion

### Principal Findings

The *¡Hola Bebé, Adiós Diabetes!* mHealth program was designed to overcome access barriers to T2DM prevention support among Hispanic women with prior GDM. Pilot testing indicated that it was well accepted, usable, and showed preliminary effectiveness at increasing self-efficacy for both physical activity and healthy eating. Weight decreased over the 8-week period but not significantly.

Interventions delivered through apps have great potential to fill the gap experienced by individuals seeking care across a range of conditions. A classification scheme for analyzing apps for preventing and managing disease proposes three dimensions for analysis: health condition (physical versus mental); prevention versus management; and, according to Green and Kreuter’s [35] Precede-Proceed Model, predisposing, enabling, and motivating factors [36]. Using this classification scheme, Hola Bebé, addresses a physical condition, that of GDM, for the prevention of type 2 diabetes, and includes factors related to all aspects of the model, such as predisposing (eg, educational audiovisual modules, healthy recipes), enabling (eg, MyPlate demonstrations, action plans, weight tracking, and badges), and motivational (motivational text messages, sharing experiences/recipes, and asking questions through a community forum).

Hola Bebé has the potential to fill the gap in care experienced by women with GDM following pregnancy. Over 86% of women with GDM have no contact with primary care in the first year post delivery, and close to 60% have no contact at 3 years post delivery [37]. This is despite recommendations from the American College of Obstetricians and Gynecologists [38] for referral to primary care and counseling for lifestyle modification in nutrition and exercise for women with a prior pregnancy complicated by GDM. Some have characterized women with prior GDM as falling into a “healthcare chasm” [39]; alternatively, others have referred to this more positively as “a fixable gap in women’s preventative healthcare” [37], which app technology can potentially address. A major advantage of an app-delivered program for Hispanic women is the widespread use of apps by this population [40], which experiences significant disparities in health care [10]. Additional strengths of using an app for behavior change include easy access potential for integration with other apps that commonly come with smartphones (eg, pedometer and music apps); faster speed, as data are stored on the smartphone; and the ease of receiving notifications.

Importantly, Hola Bebé takes advantage of the “window of opportunity” following a complicated pregnancy by bridging the gap in care through lifestyle counseling without dependency on visits to the health center and clinician [37]. This app also overcomes many barriers experienced by women who have young children at home and competing priorities for time; it can be used at home or work or while traveling, day or night, and in small doses whenever users have a few minutes. In addition, this app was culturally and linguistically tailored for Hispanic women and was developed first in Spanish. Finally, the app was designed through an iterative approach incorporating feedback from Hispanic women with recent gestational diabetes at several stages of development.

### Limitations

Given the nature of the pilot study, we were limited by a small sample size, lack of a control group, and short study duration. A further limitation was the unavailability of the app for iOS users.

### Conclusions

The widespread use of apps among Hispanic women of childbearing age holds promise for this particularly high-risk and underserved population to reduce risk factors for diabetes. This app-delivered program should be tested in a randomized controlled trial and be developed for iOS users.

## Abbreviations

CHC: community health center
DPP: Diabetes Prevention Program
GDM: gestational diabetes mellitus
mHealth: mobile health
SCT: social cognitive theory
T2DM: type 2 diabetes mellitus
